# Longitudinal trajectories of cognitive decline and temporal lobe atrophy based on baseline gonadotropins and testosterone

**DOI:** 10.3389/fnins.2025.1696274

**Published:** 2026-01-13

**Authors:** Min Zhao, Jiwei Jiang, Linlin Wang, Shiyi Yang, Wenyi Li, Qiwei Ren, Huiying Zhang, Tianlin Jiang, Shirui Jiang, Junya Zhou, Jun Xu

**Affiliations:** 1 Department of Neurology, Beijing Tiantan Hospital, Capital Medical University, Beijing, China; 2 Beijing Tiantan Hospital, China National Clinical Research Center for Neurological Diseases, Capital Medical University, Beijing, China; 3 Beijing Tiantan Hospital, Health Management Center, Capital Medical University, Beijing, China; 4 Department of Neurology, Beijing Friendship Hospital, Capital Medical University, Beijing, China; 5 Department of Critical Care Medicine, Sichuan Academy of Medical Sciences and Sichuan Provincial People’s Hospital, Affiliated Hospital of University of Electronic Science and Technology of China, Chengdu, China

**Keywords:** Alzheimer’s disease, *APOE*, cognition, gonadotropin, sex differences, temporal lobe atrophy, testosterone

## Abstract

**Introduction:**

Although previous studies have reported associations between gonadotropins, testosterone, and Alzheimer’s disease (AD), their longitudinal relationships with cognitive decline and temporal lobe atrophy remain insufficiently characterized. This study examined the association between baseline hormone levels and cognitive decline and temporal lobe volume loss trajectories, and whether these associations vary by sex or *APOE*ε*4* status.

**Methods:**

This study included 490 participants (378 MCI/112 AD; 311 men/179 women; mean age = 75.01 ± 7.52) from the Alzheimer’s Disease Neuroimaging Initiative (ADNI) cohort. Baseline plasma levels of gonadotropins (FSH, LH) and total testosterone (TT) were measured using Luminex xMAP multiplex immunoassay. Cognitive decline was assessed longitudinally through MMSE and ADAS-Cog 13 scores. Temporal lobe atrophy was quantified using tensor-based morphometry of 1.5T MRI scans, with bilateral temporal lobe volumes scaled to a normalized reference (1,000 = baseline). Linear mixed effects models were employed to relate baseline plasma hormones to longitudinal cognitive performance and temporal lobe volume.

**Results:**

Longitudinal analyses showed that higher baseline FSH levels were associated with faster cognitive decline (MMSE: β = −0.025, *P* = 0.012; ADAS-cog: β = 0.066, *P* = 0.020) and accelerated temporal lobe atrophy (β = −0.115, *P* = 0.005) in fully adjusted models. LH was also associated with faster temporal lobe atrophy (β = −0.077, *P* = 0.034), while TT showed no significant association. Sex-stratified analyses showed that higher TT was associated with slower MMSE decline in women (β = 0.022, *P* = 0.032) but not in men (*P* = 0.762), with a significant sex interaction (*P*-interaction = 0.020). Modification effects of *APOE*ε*4* status on cognition and temporal lobe volume changes were not observed for FSH, LH, or TT.

**Discussion:**

The results indicate that in individuals across the AD spectrum, elevated gonadotropin levels may exert deleterious, domain-specific effects on cognitive decline or temporal lobe atrophy. Women with lower TT levels may experience faster cognitive progression. Although future studies incorporating additional longitudinal hormone measurements and cognitive trajectories are warranted, our results underscore the importance of gonadotropins and testosterone in AD progression.

## 1 Introduction

Alzheimer’s disease (AD) is a global health challenge with increasing prevalence in the aged population (Ferretti et al., 2018; Nebel et al., 2018; Huque et al., 2023; Castro-Aldrete et al., 2025). It imposes a significant psychological and economic burden on affected individuals, families, and society. Epidemiological studies have shown that women comprise approximately two-thirds of the population with AD (Andersen et al., 1999; Satizabal et al., 2016), and they have a twofold higher lifetime risk of developing AD (Fisher et al., 2018) and a broader spectrum of dementia-related symptoms than men (Koran et al., 2017). These differences may originate from biological factors (e.g., chromosomal, epigenetic, or hormonal variations) or psychosocial–cultural factors (e.g., educational access, gender disparities) (Castro-Aldrete et al., 2025).

With advancing age, women experience a progressive arrest of reproductive function and the onset of menopause, which is characterized by the depletion of ovarian oocytes and profound changes in the hypothalamic–pituitary–gonadal (HPG) axis (Sauvé et al., 2024). In this context, estrogen deficiency has been suggested as a possible cause of the disease, indicating that hormone replacement therapy may reduce dementia risk (Nelson et al., 2002; Zandi et al., 2002). Nevertheless, data on hormone replacement therapy have been mixed—showing improvement, no change, or even a worsening of the cognition (Zandi et al., 2002; Viña and Lloret, 2010; O’Brien et al., 2014; Matyi et al., 2019). Beyond the widely discussed issue of treatment-timing windows, these contradictory results also suggest that additional factors may contribute to the increased disease risk observed in women (Matyi et al., 2019).

Luteinizing hormone (LH) and follicle-stimulating hormone (FSH) are other hormones affected by the menopausal process (Sauvé et al., 2024). As early as two decades ago, evidence began to link elevated levels of LH and FSH to AD diagnosis and cognitive decline (Bowen et al., 2000; Short et al., 2001; Casadesus et al., 2005; Meethal et al., 2005). In 2004, researchers first demonstrated that elevated serum LH could promote the secretion and deposition of Aβ in the aging brain (Bowen et al., 2004). Subsequent studies revealed that serum LH levels might be correlated with plasma Aβ concentrations (Verdile et al., 2014). Cohort studies further showed that serum FSH levels were correlated with cognitive function (Oh et al., 2025; Zhu et al., 2025). More recent experimental work has deepened this understanding. Specifically, FSH has been shown to act directly on hippocampal and cortical neurons to activate the C/EBPβ/δ-secretase pathway, thereby accelerating amyloid and Tau pathology (Xiong et al., 2022; Xiong et al., 2023). Similarly, LH overexpression or exogenous LH administration has been found to exacerbate Aβ deposition, neuronal injury, and behavioral deficits in AD mouse models (Casadesus et al., 2007; Berry et al., 2008; Jia et al., 2024). In a clinical trial, the suppression of gonadotropins was reported as a therapeutic approach that could halt cognitive decline in AD patients (Bowen et al., 2015). These study results support the biological plausibility of gonadotropins contributing to AD-related neurodegeneration. Despite these accumulated findings, current clinical research still primarily relies on cross-sectional analyses. The longitudinal effects of gonadotropins on cognitive trajectories and brain structural changes remain insufficiently investigated.

Testosterone, among the major sex hormones in men, has been found in experimental studies to exert neuroprotective effects by scavenging free radicals, improving synaptic signaling, and enhancing mitochondrial function (Grimm et al., 2014; Jia et al., 2016; Yan et al., 2019; Bianchi, 2022). Total testosterone (TT), the main indicator of testosterone, can be detected in blood. Recent population-based studies have found an association between baseline TT and the risk of cognitive decline in older men (Tang et al., 2024). Notably, current investigations of testosterone have predominantly excluded female cohorts, and whether it modulates longitudinal cognitive trajectories or neuroanatomical progression across the AD continuum remains insufficiently characterized.

In summary, although associations between gonadotropins, testosterone, and AD have been made, their longitudinal effects on cognitive decline and temporal lobe atrophy—two key markers of disease progression—remain insufficiently characterized. Moreover, whether these associations vary by sex or *APOE*ε*4* genotype has yet to be clarified. This study aimed to examine the relationships of baseline FSH, LH, and TT with cognitive decline and temporal lobe atrophy across the AD continuum and to evaluate the modifying effects of sex and *APOE*ε*4* status on these associations.

## 2 Materials and methods

### 2.1 Study participants

**FIGURE 1:**
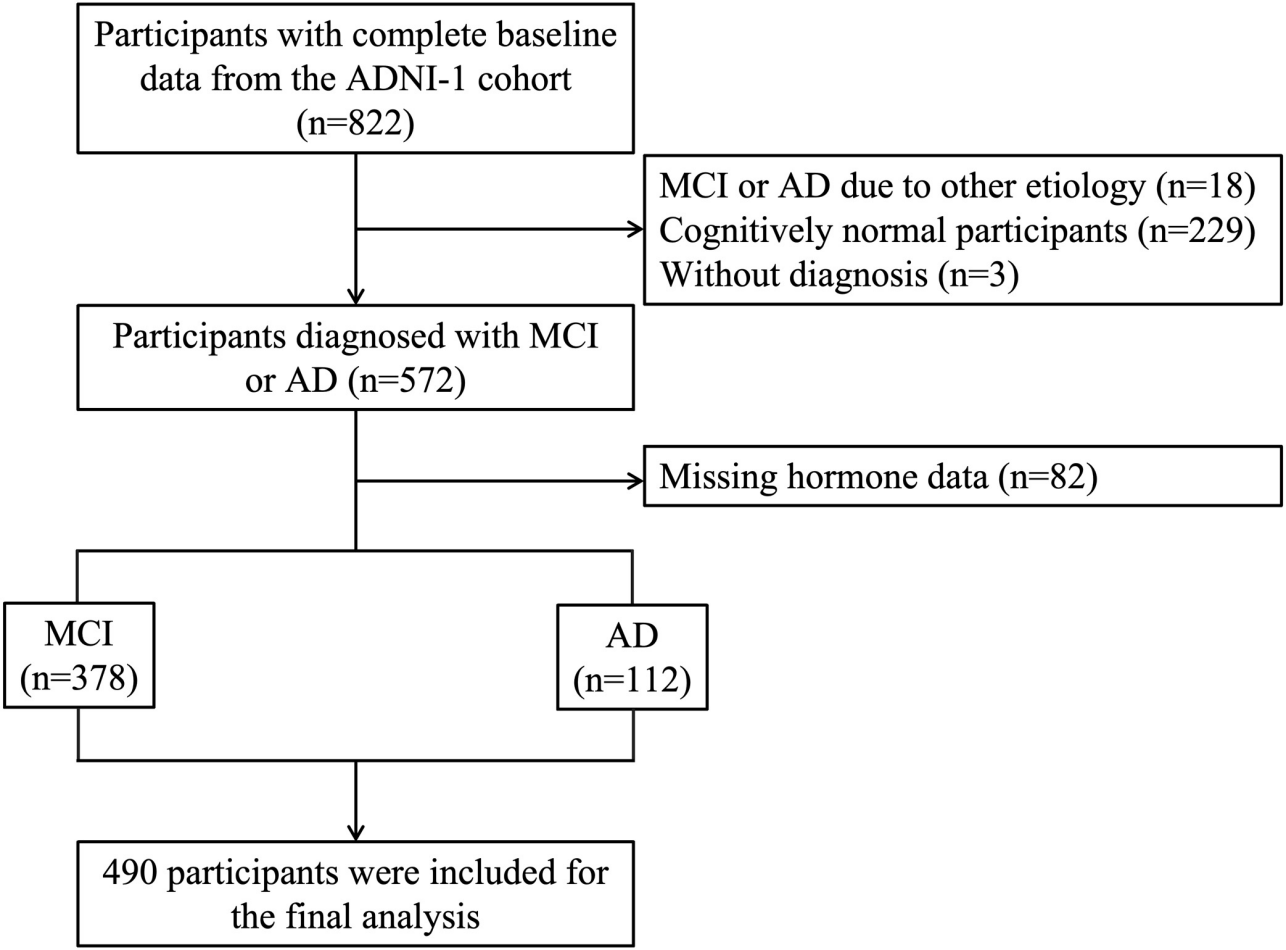
A flowchart of the inclusion and exclusion processes. ADNI, Alzheimer’s Disease Neuroimaging Initiative; MCI, mild cognitive impairment; AD, Alzheimer’s dementia.

Data were obtained from the Alzheimer’s Disease Neuroimaging Initiative (ADNI) database on 30 December 2024. ADNI was launched in 2004 as a longitudinal observational study of aging that enrolls participants diagnosed as cognitively normal (CN), subjective memory concerns (SMC), mild cognitive impairment (MCI) (both early and late stages), and AD dementia. All ADNI data used in this analysis are publicly available online. For up-to-date information, see www.adni-info.org. Written informed consent was obtained from all participants. Site-specific institutional review boards approved the ADNI protocol. Our final analysis included 490 individuals with available hormone measurements and complete clinical information (average age 75.01 ± 7.52 years; 311 men/179 women), comprising 378 patients with MCI and 112 patients with AD at baseline. A flowchart of the participant selection process is shown in Figure 1. Diagnoses were made by ADNI based on criteria described in the ADNI1 procedure manual.^1^

### 2.2 Cognitive testing and genotyping

Cognitive decline was measured as change in the widely used the Mini-Mental State Examination (MMSE) and the 13-item Alzheimer’s Disease Assessment Scale–Cognitive Subscale (ADAS-Cog 13). The ABI 7900 real-time thermo-cycler (Applied Biosystems) was used to determine the APOE genotype of ADNI participants. TaqMan quantitative PCR was applied to DNA prepared from EDTA whole blood (Quesnel et al., 2024). We characterized participants as being “0” (i.e., zero *APOE*ε*4* alleles) or “1” (i.e., one or two *APOE*ε*4* alleles).

### 2.3 Plasma hormone

Plasma samples were drawn from the subjects after fasting overnight at the baseline visit, and 190 analytes that have been reported to be altered in cancer, cardiovascular disease, metabolic disorders, inflammation and AD were measured with the Human Discovery Map panel, a multiplex immunoassay panel developed on the Luminex xMAP platform by Rules Based Medicine. The analysis was performed using plasma gonadotropin (FSH, LH), and sex hormone (TT). All participants had FSH values within the detectable range. 4 cases had LH recorded as “LOW” and 18 cases had TT recorded as “LOW.” Values recorded as “LOW” will be imputed as LLD/2.

### 2.4 Cumulative temporal lobe atrophy

The data in this section is based on a dataset developed by Hua et al. (2013) at the Laboratory of Neuro Imaging UCLA School of Medicine (Cross-sectional and longitudinal tensor-based morphometry Version 2.0). MR scans (1.5T) were downloaded from the Alzheimer’s Disease Neuroimaging Initiative (ADNI) public database (see text footnote 1). Downloaded images had been processed with the standard Mayo Clinic processing pipeline, identified with the term “scaled” (ADNI-1) in the file name. Methods for the improved longitudinal TBM (Hua et al., 2013) are as described before.

To derive a single-number summary of the 3D map of brain atrophy for each subject, a single numerical measure was derived by computing an average within a region-of-interest (ROI). The anatomically-defined ROI (stat-ROI) was used. The temporal lobe ROI (temp-ROI), including the temporal lobes of both brain hemispheres, was manually delineated on the MDT template by a trained anatomist using the Brainsuite software (version 2.11) (Shattuck and Leahy, 2002). Numerical of cumulative temporal lobe atrophy average within an anatomically defined region-of-interest including bilateral temporal lobes were selected, in which summaries are scaled by 1,000 (e.g., 1,000: no change, 1,200: 20% increase, 800: 20% loss). We ultimately included 447 subjects with at least 6, 12, 18, or 24 months of follow-up data (283 men/164 women, 345 MCI/102 AD at baseline).

### 2.5 Statistical analyses

In this study, quantitative variables are reported as mean ± standard deviation (SD) or median and interquartile range (IQR). Contingent upon the data distribution while representing the qualitative variables as counts and proportions. Analysis of continuous variables adhering to normal distribution utilized the *t*-test, whereas the Mann-Whitney U test was applied for variables diverging from normality. Categorical variables were assessed using the chi-square test or Fisher’s exact test (when expected frequencies were <5).

The Linear mixed effects (LME) models (“nlme” package) were to examine the interactive effects of baseline plasma hormone levels on longitudinal changes in cognitive trajectories (outcomes included MMSE and ADAS-cog) and cumulative temporal lobe atrophy over time. Time was operationalized as years from baseline assessment (up to 2 years of follow-up). The LME model included random intercepts and slopes. A progressive modeling approach was used. The first model (Model 1) investigated the crude, unadjusted association. Model 2 included adjustment for age, sex, education, *APOE*ε*4* status, BMI, and time-covariate interactions. The fully adjusted model (Model 3) included additional adjustments for history of hypertension, diabetes, hyperlipidemia, stroke, and time-covariate interactions. Secondary analyses examined the modifying effect of sex and *APOE*ε*4* status on the association of plasma hormones (FSH, LH, and TT) with cumulative temporal lobe atrophy and cognitive trajectories. All hormones were standardized (mean = 0 and standard deviation = 1) and time was mean centered. Corrections for multiple comparisons were made using the false discovery rate (FDR) method as an additional conservative adjustment (Benjamini et al., 1995). FDR-adjusted *P*-values were computed by grouping *P*-values within each unique combination of predictor and model type. FDR-adjusted *P*-values are provided in the results tables for reference. Because some borderline findings may be due to chance, these results should be interpreted with caution. Statistical analysis was done using R version 4.0.0. Significance was determined at *P* < 0.05 (two-tailed).

## 3 Results

### 3.1 Demographics

See Table 1 for demographics. In our sample of 490 participants, there were 179 (36.53%) women and 311 (63.47%) men (self-reported sex assigned at birth). Across all of the participants, 378 (77.14%) had been diagnosed with MCI, and 112 (22.86%) had been diagnosed with AD. Participants had a mean age of 75.01 years and a median of 16 years of education. Women in our sample were younger, had fewer years of education, and higher levels of FSH and LH. Men had higher levels of MMSE and TT. In the diagnostic stratification group, we observed no significant sex differences in baseline FSH, LH, and TT (Table 1).

**TABLE 1:** Participant characteristics.

Baseline characteristics		Full analytic sample	Men	Women	*t/Z/*χ*2*	*P*-value	MCI	AD	*t/Z/*χ*2*	*P*-value
**Outcomes**										
Diagnosis	0	378 (77.14)	246 (79.10)	132 (73.74)	1.849	0.174	(-)	(-)	(-)	(-)
	1	112 (22.86)	65 (20.90)	47 (29.26)	–	–	(-)	(-)	–	–
Sex	Men	311 (63.47)	(-)	(-)	(-)	(-)	246 (65.08)	65 (58.04)	1.849	0.174
	Women	179 (36.53)	(-)	(-)	–	–	132 (34.92)	47 (41.96)	–	–
Age		75.01 ± 7.52	75.52 ± 7.34	74.13 ± 7.77	1.969	0.099	75.04 ± 7.37	74.91 ± 8.05	0.162	0.871
Education		16 (13–18)	16 (14–18)	16 (12–18)	−3.457	<0.001*	16 (13.75–18)	16 (13–18)	−1.602	0.109
BMI		25.93 ± 3.84	26.42 ± 3.62	25.09 ± 4.06	3.711	<0.001*	26.03 ± 3.84	25.62 ± 3.81	0.981	0.327
*APOE*ε*4* status	Negative	223 (45.51)	143 (45.98)	80 (44.69)	0.076	0.783	185 (48.94)	38 (33.93)	7.853	0.005*
	Positive	267 (54.49)	168 (54.02)	99 (55.31)	–	–	193 (51.06)	74 (66.07)	–	–
Hypertension	0	254 (51.84)	159 (51.13)	95 (53.07)	1.730	0.678	196 (51.85)	58 (51.79)	<0.001	0.99
	1	236 (48.16)	152 (48.87)	84 (46.93)	–	–	182 (48.15)	54 (48.21)	–	–
Diabetes	0	454 (72.65)	283 (91.00)	171 (95.53)	3.431	0.064	349 (92.33)	105 (93.75)	0.257	0.612
	1	36 (7.35)	28 (9.00)	8 (4.47)	–	–	29 (7.67)	7 (6.25)	–	–
Hyperlipidemia	0	221 (45.10)	137 (44.05)	84 (46.93)	0.380	0.538	170 (44.97)	51 (45.54)	0.011	0.916
	1	269 (54.90)	174 (55.95)	95 (53.07)	–	–	208 (55.03)	61 (54.46)	–	–
Stroke	0	480 (97.96)	304 (97.75)	176 (98.32)	0.188	0.665	370 (97.88)	110 (98.21)	0.047	0.828
	1	10 (2.04)	7 (2.25)	3 (1.68)	–	–	8 (2.12)	2 (1.79)	–	–
MMSE	–	26 (25–28)	26 (25–28)	26 (24–28)	−2.232	0.026*	27 (26–29)	24 (22–25)	−12.860	<0.001*
ADAS-cog 13	–	20.33 (15.67, 25.33)	19.67 (15.92, 24.00)	21.67 (15.67, 26.33)	−1.863	0.062	18.33 (14.58, 23.00)	28.34 (22.41, 33.83)	−10.580	<0.001*
FSH (mIU/mL)	–	13 (5.30–41)	6.4 (3.70–11.00)	47 (35.00–59.00)	−17.590	<0.001*	12 (5.3–38)	14 (5.18–49.5)	−0.875	0.382
LH (mIU/mL)	–	4.8 (2.20–11.00)	2.6 (1.80–4.40)	12.00 (9.00–16.00)	−16.741	<0.001*	4.8 (2.3–11)	4.7 (1.91–1.75)	−0.720	0.472
TT (ng/ml)	–	2.1 (0.83–3.20)	2.9 (2.10–3.5)	0.62 (0.41–0.93)	−17.479	<0.001*	2.1 (0.75–3.20)	1.85 (0.80–3.20)	−0.967	0.333

*APOE* ε*4*, apolipoprotein E ε4 allele; MMSE, Mini-Mental State Examination; ADAS-cog 13, 13-item Alzheimer’s Disease Assessment Scale-Cognitive Subscale; FSH, follicle-stimulating hormone; LH, luteinizing hormone; TT, total testosterone.

*Remains *P* < 0.05.

### 3.2 Baseline plasma hormones and cognitive trajectories

**FIGURE 2:**
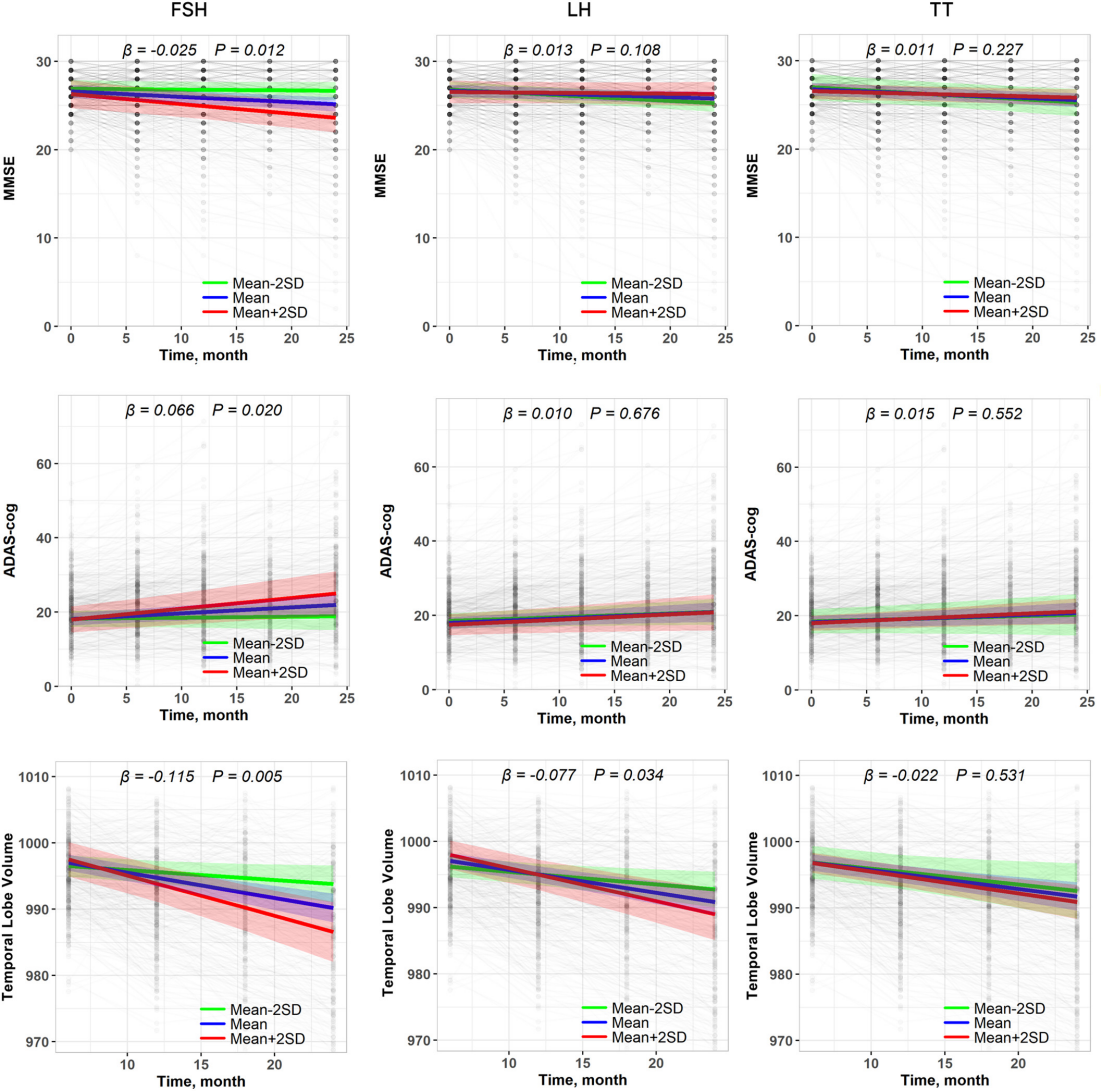
Association of baseline plasma hormones with longitudinal changes in cognitive function and temporal lobe volume. Results are derived from linear mixed effect models adjusted for baseline age, sex, education, *APOE* ε*4* status, hypertension, diabetes, hyperlipidemia, stroke, and the interaction between covariates and time. Estimates, derived from the plasma hormones × time interaction terms, represent the difference in annual change in cognitive scores or temporal lobe volume (β estimates) per standard deviation (SD) increase in baseline hormone.

Longitudinal analysis showed that a higher baseline FSH level was associated with faster cognitive decline in fully adjusted models (Model 3) (MMSE: β = −0.025, *P* = 0.012; ADAS-cog: β = 0.066, *P* = 0.020). LH and TT were initially associated with cognitive changes in univariate models but lost significance after covariate adjustment (Model 3, *P* > 0.05) (Figure 2 and Table 2). The results were similar in sensitivity analyses that did not adjust for medical history (Model 2) (Table 2).

**TABLE 2:** Associations between baseline plasma hormones and longitudinal changes in cognitive trajectories.

Hormones	Outcomes	Model 1				Model 2				Model 3 (fully adjusted model)			
		β	95% CI	*P*	FDR-*P*	β	95%CI	*P*	FDR-*P*	β	95% CI	*P*	FDR-*P*
FSH	MMSE	−0.023	(−0.034, −0.012)	<0.001*	<0.001	−0.025	(−0.045, −0.006)	0.012*	0.036	−0.025	(−0.045, −0.005)	0.012*	0.036
LH	MMSE	−0.006	(−0.017, 0.006)	0.331	0.331	0.013	(−0.003, 0.029)	0.116	0.174	0.013	(−0.003, 0.030)	0.108	0.162
TT	MMSE	0.017	(0.006, 0.027)	0.003*	0.005	0.011	(−0.006, 0.028)	0.189	0.189	0.011	(−0.007, 0.028)	0.227	0.227
**Hormones**	**Outcomes**	**β**	**95% CI**	***P***	**FDR-*P***	**β**	**95%CI**	***P***	**FDR-*P***	**β**	**95% CI**	***P***	**FDR-*P***
FSH	ADAS-cog 13	0.073	(0.042, 0.104)	<0.001*	<0.001	0.066	(0.011, 0.121)	0.019*	0.057	0.066	(0.010, 0.121)	0.020*	0.060
LH	ADAS-cog 13	0.047	(0.016, 0.079)	0.004*	0.006	0.008	(−0.037, 0.053)	0.723	0.723	0.010	(−0.036, 0.055)	0.676	0.676
TT	ADAS-cog 13	−0.042	(−0.074, −0.011)	0.008*	0.008	0.013	(−0.036, 0.061)	0.607	0.911	0.015	(−0.034, 0.064)	0.552	0.828

Results are derived from linear mixed effect models. Model 1, univariate model; Model 2, adjusted for baseline age, sex, education, apolipoprotein E ε4 allele (*APOE* ε*4*) status, BMI, and time-covariate interactions; Model 3, additionally adjusted for hypertension, diabetes, hyperlipidemia, stroke and time-covariate interactions. Longitudinal β values reflect the difference in monthly change in cognitive scores per SD increase in hormone. longitudinal effects reflect plasma hormone × time interactions derived from the same model.

*Remains *P* < 0.05. MMSE, Mini-Mental State Examination; ADAS-cog 13, 13-item Alzheimer’s Disease Assessment Scale-Cognitive Subscale; FSH, follicle-stimulating hormone; LH, luteinizing hormone; TT: total testosterone.

**FIGURE 3:**
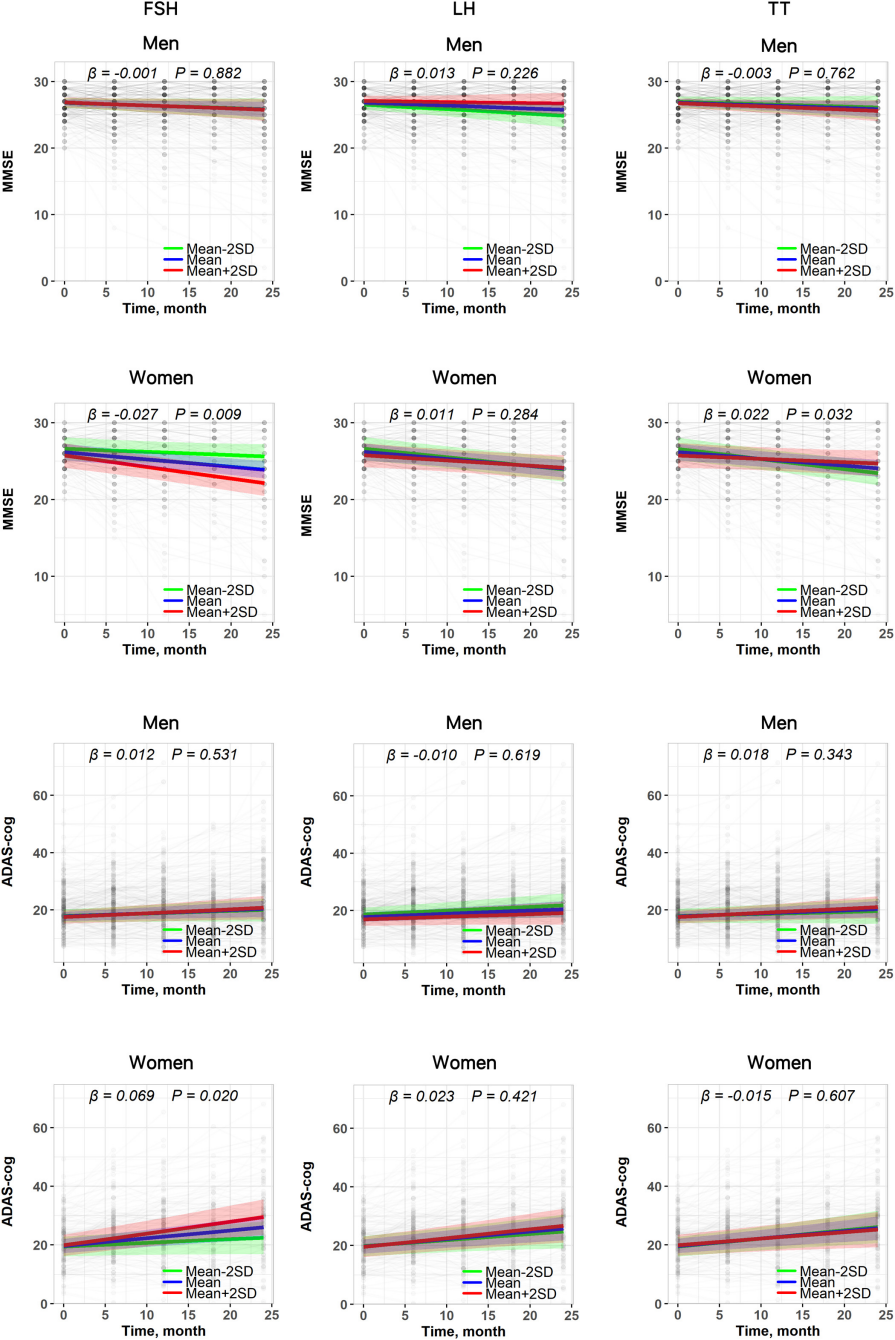
The relationship between baseline plasma hormones and longitudinal changes in cognitive trajectories in both sexes. Results are derived from linear mixed effect models adjusted for baseline age, sex, education, *APOE* ε*4* status, hypertension, diabetes, hyperlipidemia, stroke, and the interaction between covariates and time. Estimates, derived from the plasma hormones × time interaction terms, represent the difference in annual change in cognitive scores (β estimates) per standard deviation (SD) increase in baseline hormone.

Examination of effect modification by sex revealed an isolated TT × sex interaction on longitudinal change in cognitive trajectories (*P*-interaction = 0.032). Sex stratification analysis showed that higher baseline TT was associated with slower MMSE decline in women (β = 0.022, *P* = 0.032) but not in men (β = −0.003, *P* = 0.762). Sex did not modify the association of FSH and LH with brain volume change. Although a stronger association between FSH and cognitive trajectories was observed in women (MMSE: β = −0.027, *P* = 0.009; ADAS-cog: β = 0.069, *P* = 0.020), the sex interaction did not reach statistical significance (MMSE: *P*-interaction = 0.503; ADAS-cog: *P*-interaction = 0.874) (Figure 3 and Table 3). Moreover, the *APOE*ε*4* status did not modify the association of FSH, LH and TT with cognitive trajectories (Table 4).

**TABLE 3:** The effects of sex on the relationship between baseline plasma hormones and longitudinal changes in cognitive trajectories.

Hormones	Sex	MMSE			Interaction	Sex	ADAS-cog 13			Interaction
		β	95% CI	*P*	*P*		β	95% CI	*P*	*P*
FSH	Men	−0.001	(−0.021, 0.018)	0.882	0.503	Men	0.012	(−0.026, 0.050)	0.531	0.874
	Women	−0.027	(−0.048, −0.007)	0.009*		Women	0.069	(0.012, 0.126)	0.020*	
		**β**	**95% CI**	***P***	***P***		**β**	**95% CI**	***P***	***P***
LH	Men	0.013	(−0.008, 0.034)	0.226	0.937	Men	−0.010	(−0.051, 0.031)	0.619	0.807
	Women	0.011	(−0.009, 0.031)	0.284		Women	0.023	(−0.033, 0.079)	0.421	
		**β**	**95% CI**	***P***	***P***		**β**	**95% CI**	***P***	***P***
TT	Men	−0.003	(−0.023, 0.017)	0.762	0.020*	Men	0.018	(−0.020, 0.057)	0.343	0.302
	Women	0.022	(0.002, 0.042)	0.032*		Women	−0.015	(−0.071, 0.042)	0.607	

The interaction reflects whether the association between each plasma hormone and cognitive trajectories vary by sex. Stratified analyses were examined to explore potential trends of sex status differences (adjusted variables same as Model 3).

*Remains *P* < 0.05. MMSE, Mini-Mental State Examination; ADAS-cog 13, 13-item Alzheimer’s Disease Assessment Scale-Cognitive Subscale; FSH, Follicle-stimulating hormone; LH, luteinizing hormone; TT, total testosterone.

**TABLE 4:** The effects of apolipoprotein E ε4 allele (*APOE* ε*4*) status on the relationship between baseline plasma hormones and longitudinal changes in cognitive trajectories.

Hormones	*APOE*ε4 status	MMSE			Interaction	*APOE*ε4 status	ADAS-cog 13			Interaction
		β	95% CI	*P*	*P*		β	95% CI	*P*	*P*
FSH	Negative	−0.036	(−0.063, −0.008)	0.012*	0.453	Negative	0.046	(−0.029, 0.122)	0.231	0.083
	Positive	−0.010	(−0.051, 0.031)	0.624		Positive	0.067	(−0.014, 0.148)	0.108	
		**β**	**95% CI**	***P***	***P***		**β**	**95% CI**	***P***	***P***
LH	Negative	0.003	(−0.019, 0.025)	0.789	0.218	Negative	0.007	(−0.052, 0.067)	0.812	0.284
	Positive	0.024	(−0.010, 0.058)	0.176		Positive	0.003	(−0.066, 0.072)	0.941	
		**β**	**95% CI**	***P***	***P***		**β**	**95% CI**	***P***	***P***
TT	Negative	0.018	(−0.005, 0.042)	0.128	0.479	Negative	0.003	(−0.062, 0.068)	0.931	0.203
	Positive	−0.007	(−0.043, 0.030)	0.712		Positive	0.032	(−0.041, 0.105)	0.396	

The interaction reflects whether the association between each plasma hormone and cognitive trajectories vary by *APOE*ε4 status. Stratified analyses were examined to explore potential trends of *APOE*ε4 status differences (adjusted variables same as Model 3).

*Remains *P* < 0.05. FSH, MMSE: Mini-Mental State Examination; ADAS-cog 13, 13-item Alzheimer’s Disease Assessment Scale-Cognitive Subscale; FSH, follicle-stimulating hormone; LH, luteinizing hormone; TT, total testosterone.

### 3.3 Baseline plasma hormones and cumulative temporal lobe atrophy

Higher baseline FSH and LH levels were associated with faster progression of temporal lobe atrophy in fully adjusted models (FSH: β = −0.115, *P* = 0.005; LH: β = −0.077, *P* = 0.034). TT showed no significant associations with atrophy rates (Figure 2 and Table 5). The results were similar in sensitivity analyses that did not adjust for medical history (Model 2) (Table 5).

**TABLE 5:** Associations between baseline plasma hormones and longitudinal changes in temporal lobe volume.

Hormones	Model 1				Model 2				Model 3			
	β	95% CI	*P*	FDR-*P*	β	95%CI	*P*	FDR-*P*	β	95% CI	*P*	FDR-*P*
FSH	−0.107	(−0.154, −0.060)	<0.001*	<0.001	−0.120	(−0.200, −0.041)	0.003*	0.009	−0.115	(−0.194, −0.036)	0.005*	0.015
	**β**	**95% CI**	***P***	**FDR-*P***	**β**	**95%CI**	***P***	**FDR-*P***	**β**	**95% CI**	***P***	**FDR-*P***
LH	−0.096	(−0.145, −0.047)	<0.001*	<0.001	−0.081	(−0.151, −0.011)	0.023*	0.035	−0.077	(−0.147, −0.006)	0.034*	0.051
	**β**	**95% CI**	***P***	**FDR-*P***	**β**	**95%CI**	***P***	**FDR-*P***	**β**	**95% CI**	***P***	**FDR-*P***
TT	0.042	(−0.004, 0.088)	0.075	0.075	−0.027	(−0.095, 0.041)	0.444	0.444	−0.022	(−0.090, 0.046)	0.531	0.531

Results are derived from linear mixed effect models. Model 1, univariate model; Model 2, adjusted for baseline age, sex, education, *APOE* ε*4* status, BMI, and time-covariate interactions; Model 3, additionally adjusted for hypertension, diabetes, hyperlipidemia, stroke and time-covariate interactions. Longitudinal β values reflect the difference in monthly change in temporal lobe atrophy per SD increase in hormone. longitudinal effects reflect plasma hormone × time interactions derived from the same model. Numerical of cumulative temporal lobe atrophy average within an anatomically defined region-of-interest including bilateral temporal lobes were selected, in which summaries are scaled by 1,000 (e.g., 1,000: no change, 1,200: 20% increase, 800: 20% loss).

*Remains *P* < 0.05. FSH, follicle-stimulating hormone; LH, luteinizing hormone; TT, total testosterone.

**FIGURE 4:**
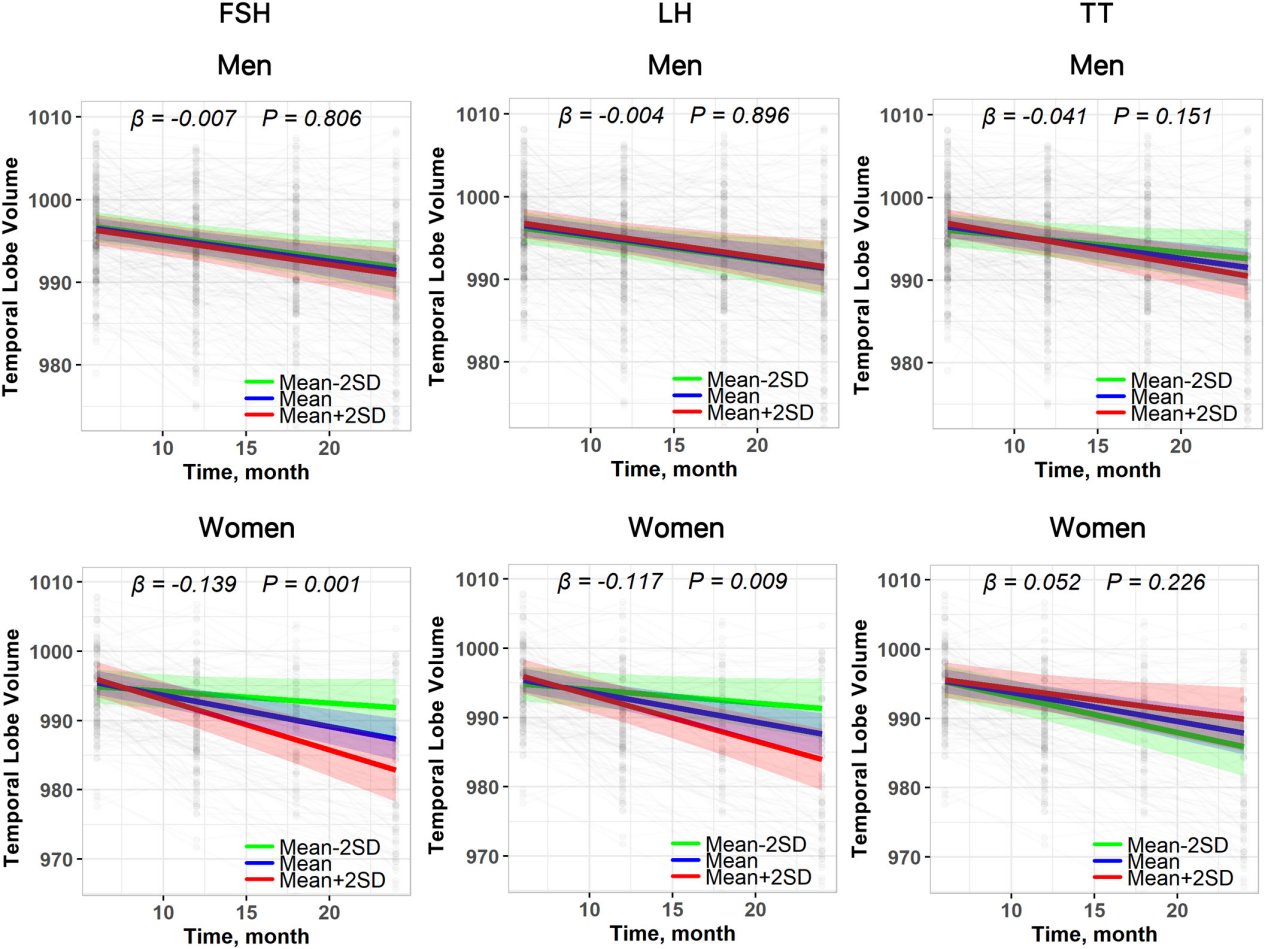
The relationship between baseline plasma hormones and longitudinal changes in temporal lobe volume in both sexes. Results are derived from linear mixed effect models adjusted for baseline age, sex, education, *APOE* ε*4* status, hypertension, diabetes, hyperlipidemia, stroke, and the interaction between covariates and time. Estimates, derived from the plasma hormones × time interaction terms, represent the difference in annual change in temporal lobe volume (β estimates) per standard deviation (SD) increase in baseline hormone.

Subgroup analysis revealed that the association of FSH and LH with faster declines in temporal lobe volume was more significant in women than in men (Figure 4 and Table 6), and the relationship between FSH and faster temporal lobe volume loss was more significant in *APOE*ε*4*-negative participants. However, modification effects of sex and *APOE*ε*4* status on temporal lobe volume changes were not observed for FSH, LH, or TT (Table 6).

**TABLE 6:** The effects of sex or apolipoprotein E ε4 allele (*APOE* ε*4*) status on the relationship between baseline plasma hormones and longitudinal changes in temporal lobe volume.

Hormones	Sex				Interaction	*APOE*ε4 status				Interaction
		β	95% CI	*P*	*P*		β	95% CI	*P*	*P*
FSH	Men	−0.007	(−0.064, 0.050)	0.806	0.223	Negative	−0.087	(−0.215, 0.041)	0.183	0.880
	Women	−0.139	(−0.223, −0.056)	0.001*		Positive	−0.133	(−0.234, −0.031)	0.011*	
		**β**	**95% CI**	***P***	***P***		**β**	**95% CI**	***P***	***P***
LH	Men	−0.004	(−0.064, 0.056)	0.896	0.350	Negative	−0.084	(−0.199, 0.031)	0.153	0.876
	Women	−0.117	(−0.204, −0.031)	0.009*		Positive	−0.063	(−0.153, 0.027)	0.171	
		**β**	**95% CI**	***P***	***P***		**β**	**95% CI**	***P***	***P***
TT	Men	−0.041	(−0.098, 0.015)	0.151	0.366	Negative	0.046	(−0.061, 0.153)	0.400	0.144
	Women	0.052	(−0.031, 0.135)	0.226		Positive	−0.085	(−0.176, 0.007)	0.071	

The interaction reflects whether the association between each plasma hormone and temporal lobe atrophy vary by sex or *APOE*ε4 status. Stratified analyses were examined to explore potential trends of sex or *APOE*ε4 status differences (adjusted variables same as Model 3). Numerical of cumulative temporal lobe atrophy average within an anatomically defined region-of-interest including bilateral temporal lobes were selected, in which summaries are scaled by 1,000 (e.g., 1,000: no change, 1,200: 20% increase, 800: 20% loss).

*Remains *P* < 0.05. FSH, follicle-stimulating hormone; LH, luteinizing hormone; TT, total testosterone.

## 4 Discussion

The present study examined whether baseline plasma gonadotropins (FSH and LH) and sex hormone (TT) predict longitudinal changes in cognitive performance and temporal lobe volume in individuals with MCI and AD. We found that elevated baseline plasma FSH levels could accelerate cognitive decline and temporal lobe atrophy, and that higher baseline plasma LH levels could accelerate temporal lobe volume loss. These findings were more significant in women. Although plasma TT was not associated with cognitive and temporal lobe volumetric changes, we observed a potential sex-specific interaction effect between baseline TT and cognitive performance (MMSE score). These results provide important insights for assessing the risk of AD progression in clinical practice using baseline hormone levels. Understanding how these sex-specific hormones modulate disease progression across the AD spectrum can guide the development of more precise individualized intervention strategies.

Mounting evidence has demonstrated that FSH and FSHR perform actions outside the reproductive tract, including in the hippocampal and cortical neurons (Xiong et al., 2022), hepatocytes (Song et al., 2016; Qi et al., 2018; Guo et al., 2019), adipose tissue (Liu et al., 2015, 2017), and pancreatic islet (Cheng et al., 2023), and they play significant roles in postmenopausal diseases in females. In the present study, the finding that higher plasma FSH was associated with steeper longitudinal declines in cognitive function and temporal lobe volume aligns with previous basic research that found FSH contributed to the pathological burden of AD and subsequent cognitive impairment (Xiong et al., 2022). Moreover, sex-stratified analyses demonstrated stronger associations of FSH with cognitive decline and temporal lobe atrophy in women, along with rising FSH levels in older men (Araujo and Wittert, 2011) and systemic FSH-induced AD pathology in both sexes (Xiong et al., 2022). These findings suggest that the faster AD progression in women may be associated with higher exposure to FSH. Prior cohort studies have shown that some women experience cognitive decline during perimenopause (Meyer et al., 2003; Greendale et al., 2009; Epperson et al., 2013). Note that, at this stage, the estrogen level in women remains relatively stable, while the serum FSH begins to rise sharply (Randolph et al., 2011). Pituitary gonadotropin was first proposed as a potential mediating factor for the occurrence and development of AD 20 years ago (Bowen et al., 2000; Short et al., 2001). Nevertheless, the mechanistic connections between pituitary gonadotropins (FSH and LH) and AD pathogenesis remain unclear, and clinical evidence remains limited. Recently, a foundational study published in *Nature* demonstrated that FSH acted directly on hippocampal and cortical neurons to accelerate Aβ and Tau deposition, and that this effect was mediated by the neuronal C/EBPβ–δ-secretase pathway (Xiong et al., 2022). *APOE*ε*4* cooperates with FSH to activate the C/EBPβ/δ-secretase pathway, jointly triggering the occurrence of AD-like pathology (Xiong et al., 2023). Further, FSHR has been demonstrated to regulate intracellular cAMP levels through coupled Gαs protein in ovarian granulosa cells or Gαi protein in Sertoli cells, respectively (Simoni et al., 1997; Crépieux et al., 2001). Similarly, an *in vitro* study demonstrated that high FSH levels could activate Gαi to inhibit the cAMP/PKA pathway (Cheng et al., 2023), which has been proved to be involved in the regulation of synaptic plasticity (Shen et al., 2016) and neuropsychiatric symptoms on the AD continuum (Jiang et al., 2025). In conclusion, targeting the FSH-related pathway may provide a novel therapeutic intervention for AD (Lemprière, 2022).

In the current study, we found that higher LH was associated with faster progression of temporal lobe atrophy. LH, a pituitary gonadotropin, has also been implicated in the pathogenesis of memory deficits (Palm et al., 2014). For example, LH over-expressed mice were found to develop cognitive impairment (Casadesus et al., 2007; Berry et al., 2008). *In vivo* studies reported that the exogenous injection of LH exacerbated behavioral impairments in mice and resulted in worsened neuronal damage and increased Aβ deposition (Jia et al., 2024). In addition, increased LH levels have been detected both in the cytoplasm of pyramidal neurons and within neurofibrillary tangles in the brain of those with AD (Bowen et al., 2002). Although tremendous strides have been made in understanding the mechanism of LH in AD over the last few years, longitudinal clinical cohort studies on this topic remain insufficient. Our study aims to build upon this foundation to further investigation. However, the lack of an association between LH levels and the progression of cognitive function is unexpected, particularly in the context of the robust associations between LH and temporal lobe atrophy. Given that declines in brain volume typically precede cognitive impairment (Hansson, 2021), the selective impact of LH on temporal lobe atrophy progression may correlate with early-stage pathological alterations in AD development. A recent study reported that higher pTau-181 and GFAP were indicative of future brain volume loss in cognitively normal older adults, while Aβ_42/40_ and GFAP levels were associated with faster subsequent cognitive decline in specific cognitive domains (Dark et al., 2024). The consecutive investigations from the Australian imaging, biomarkers, and lifestyle cohort showed that the serum levels of LH were correlated with plasma Aβ_1–40_ levels in cognitively normal or older males diagnosed with MCI. These findings suggest the potential progressive involvement of LH in the early preclinical stages of AD (Verdile et al., 2008, 2014). The interaction between LH and AD biomarkers requires further investigation, and the translation of these pathological or structural changes into detectable cognitive decline may require longer-term follow-up studies. Moreover, as gonadotropins is affected by the menopausal transition, LH and FSH both trigger androgen or estrogen production and are often regulated in the same direction (Lopez-Lee et al., 2024). Thus, future studies should aim to elucidate the potential synergistic roles of FSH and LH in AD progression.

Age-related decline in circulating total testosterone (TT) concentrations parallels the increasing prevalence of geriatric comorbidities in aging men (Shi et al., 2013; Marriott et al., 2022b). For example, *in vitro* cell experiments found that treatment with testosterone increased cleavage of the β-amyloid precursor protein to enhance secretion of non-amyloidogenic fragments (Goodenough et al., 2000; Gouras et al., 2000), and that testosterone was more effective in preventing β-amyloid-induced cell death compared with estradiol (Pike, 2001; Park et al., 2007). Animal studies also demonstrated that testosterone supplementation increased spine synapses in the rat hippocampus (Leranth et al., 2003). Low testosterone levels have been proved to be associated with poorer cognitive function and a higher risk of AD (Moffat et al., 2004; Seidl and Massman, 2015; Tang et al., 2024). However, these studies were not consistent and were mainly derived from male samples (Geerlings et al., 2006), despite similar age-related testosterone decline in females (Dratva et al., 2024). Testosterone has been historically regarded as a male hormone and is often overlooked in female brain health research. In fact, although circulating levels of testosterone in women are on average one tenth of that in men (Judd et al., 1974), the levels of testosterone in the brain are similar between the two sexes (Hammond et al., 1983; Rosario et al., 2011). Only limited studies have suggested that low testosterone levels in elderly female *APOE*ε*4* carriers may exert detrimental and domain-specific effects on cognitive performance across the aging-MCI–AD continuum (Dratva et al., 2024). In the present study, we observed an association between low TT levels and accelerated MMSE score decline in women, whereas no analogous association was detected for ADAS-cog score. Besides, we observed no association between higher plasma TT abundance and accelerated decline in either cognitive performance or temporal lobe volume in the overall cohort and male subgroup. Several factors could explain why the protective effects of higher plasma TT were only observed in women, a pattern that differs from prior reports of testosterone-related benefits primarily being found in men. First, most earlier studies were conducted in cognitively normal aging men (Ford et al., 2018; Tang et al., 2024), whereas our study additionally included female participants. Moreover, all participants in our cohort had already progressed to the stages of MCI or AD. Because hormonal regulation and androgen receptor signaling may become dysregulated as AD pathology advances, testosterone’s neuroprotective effects may diminish or become more heterogeneous in symptomatic male patients. In contrast, women have inherently low circulating TT levels, so a further reduction in TT is more likely to constitute a risk factor, resulting in a more pronounced association between TT and disease progression in the middle and late stages. Second, it is now well accepted that serum testosterone levels decline progressively with aging in men (Huang et al., 2016). Most prior studies demonstrating a protective effect of TT in males were conducted in cohorts with mean ages typically ranging from 60 to 70 years (Marriott et al., 2022a; Tang et al., 2024). In contrast, participants in our cohort were substantially older, with a mean age of approximately 75 years. At this advanced age, the age-related decline in TT levels among men may have reduced the detectability of associations with cognitive trajectories. Therefore, future large cohort studies may benefit from incorporating age stratification to better disentangle the stage-dependent effects of testosterone across the aging continuum. Third, as the principal androgen (Heijboer and Hannema, 2023), testosterone exerts neuroprotective effects by facilitating the clearance of Aβ through the modulation of microglial activity (Gouras et al., 2000; Du et al., 2025), enhancing synaptic plasticity (Schulz and Korz, 2010; Yan et al., 2019), inhibiting neuroinflammation (Jayaraman et al., 2014), and improving neuronal energy metabolism. These protective effects could occur directly through pathways mediated by the androgen receptor or partially through its conversion to estradiol (Gillies and McArthur, 2010). Some studies have reported that the neuroprotective effects of testosterone can be blocked by aromatase inhibitors, suggesting that its neurobiological actions may depend, at least in part, on aromatization to estradiol. Testosterone and estradiol have been shown to share overlapping mechanisms in exerting anxiolytic and antidepressant effects (Yaffe, 2004; Carrier et al., 2015). In addition, the effects of TT in women may depend more heavily on aromatization pathways (Gillies and McArthur, 2010). These complex hormonal metabolic networks may contribute to the aforementioned differences. Future research should incorporate assessments of estradiol and investigate its interactions with testosterone and gonadotropins to achieve a more comprehensive understanding.

The study had some limitations. First, the study lacked estrogen data, which precluded an evaluation of its potential effects on the biological actions of TT and cognitive function. Future investigations should incorporate estrogen to explore the complex feedback loops within the HPG axis in AD pathogenesis. Second, the study only included baseline hormone levels and thus failed to analyze the impact of hormone dynamic changes on AD progression. Incorporating longitudinal, multi-timepoint hormone assessments in future studies will provide stronger evidence regarding their temporal relationship with AD progression and potential causal mechanisms. Third, the null finding regarding *APOE*ε*4* status suggests that gonadotropins and testosterone may affect AD pathology through mechanisms unrelated to *APOE*ε*4*. However, the homozygous sample size of *APOE*ε*4* was limited, so further verification is still needed. Finally, ADNI was a single-center study. Therefore, the current findings still need to be tested in other cohort studies to determine if the results can be generalized outside of ADNI.

## 5 Conclusion

This study elucidated the critical roles of gonadotropins (FSH and LH) and sex hormones (TT) in AD progression in older populations. We demonstrated that elevated baseline FSH levels accelerated both cognitive decline and temporal lobe atrophy, while increased LH was specifically associated with faster temporal lobe volume loss, and that these associations were more pronounced in women. Although TT did not show a significant association with cognitive or neuroimaging changes overall, we found a potential sex-specific interaction between TT and MMSE scores. In women, lower TT levels were associated with a faster decline in MMSE scores. Our results suggest that the sex-specific hormones may offer reliable targets for future interventions aimed at modifying AD progression in older adults.

## Data Availability

Publicly available datasets were analyzed in this study. This data can be found here: the datasets for this study were obtained from the Alzheimer’s Disease Neuroimaging Initiative (ADNI) database (https://adni.loni.usc.edu/).

## Footnotes

1
https://adni.loni.usc.edu/

## References

[B1] Andersen K. Launer L. J. Dewey M. E. Letenneur L. Ott A. Copeland J. R. (1999). Gender differences in the incidence of AD and vascular dementia: The EURODEM studies. EURODEM incidence research group. *Neurology* 53 1992–1997. 10.1212/wnl.53.9.1992 10599770

[B2] Araujo A. B. Wittert G. A. (2011). Endocrinology of the aging male. *Best Pract. Res. Clin. Endocrinol. Metab.* 25 303–319. 10.1016/j.beem.2010.11.004 21397200 PMC3073592

[B3] Benjamini Y. Drai D. Elmer G. Kafkafi N. Golani I. (1995). Controlling the false discovery rate: A practical and powerful approach to multiple testing. *J. R. Stat. Soc. Ser. B* 57 289–300. 10.1111/j.2517-6161.1995.tb02031

[B4] Berry A. Tomidokoro Y. Ghiso J. Thornton J. (2008). Human chorionic gonadotropin (a luteinizing hormone homologue) decreases spatial memory and increases brain amyloid-β levels in female rats. *Hormones Behav.* 54 143–152. 10.1016/j.yhbeh.2008.02.006 18413150 PMC2613844

[B5] Bianchi V. E. (2022). Impact of testosterone on Alzheimer’s disease. *World J. Mens. Health* 40 243–256. 10.5534/wjmh.210175 35021306 PMC8987133

[B6] Bowen R. L. Isley J. P. Atkinson R. L. (2000). An association of elevated serum gonadotropin concentrations and Alzheimer disease? *J. Neuroendocrinol.* 12 351–354. 10.1046/j.1365-2826.2000.00461.x 10718932

[B7] Bowen R. L. Perry G. Xiong C. Smith M. A. Atwood C. S. (2015). A clinical study of lupron depot in the treatment of women with Alzheimer’s disease: Preservation of cognitive function in patients taking an acetylcholinesterase inhibitor and treated with high dose lupron over 48 weeks. *J. Alzheimers Dis.* 44 549–560. 10.3233/jad-141626 25310993

[B8] Bowen R. L. Smith M. A. Harris P. L. R. Kubat Z. Martins R. N. Castellani R. J. (2002). Elevated luteinizing hormone expression colocalizes with neurons vulnerable to Alzheimer’s disease pathology. *J. Neurosci. Res.* 70 514–518. 10.1002/jnr.10452 12391612

[B9] Bowen R. L. Verdile G. Liu T. Parlow A. F. Perry G. Smith M. A. (2004). Luteinizing hormone, a reproductive regulator that modulates the processing of amyloid-beta precursor protein and amyloid-beta deposition. *J. Biol. Chem.* 279 20539–20545. 10.1074/jbc.M311993200 14871891

[B10] Carrier N. Saland S. K. Duclot F. He H. Mercer R. Kabbaj M. (2015). The anxiolytic and antidepressant-like effects of testosterone and estrogen in gonadectomized male rats. *Biol. Psychiatry* 78 259–269. 10.1016/j.biopsych.2014.12.024 25683735 PMC4501899

[B11] Casadesus G. Atwood C. S. Zhu X. Hartzler A. W. Webber K. M. Perry G. (2005). Evidence for the role of gonadotropin hormones in the development of Alzheimer disease. *Cell Mol. Life Sci.* 62 293–298. 10.1007/s00018-004-4384-0 15723165 PMC11924470

[B12] Casadesus G. Milliken E. L. Webber K. M. Bowen R. L. Lei Z. Rao C. V. (2007). Increases in luteinizing hormone are associated with declines in cognitive performance. *Mol. Cell. Endocrinol.* 269 107–111. 10.1016/j.mce.2006.06.013 17376589

[B13] Castro-Aldrete L. Einsiedler M. Novakova Martinkova J. Depypere H. Alvin Ang T. F. Mielke M. M. (2025). Alzheimer disease seen through the lens of sex and gender. *Nat. Rev. Neurol.* 21 235–249. 10.1038/s41582-025-01071-0 40229578

[B14] Cheng Y. Zhu H. Ren J. Wu H. Y. Yu J. E. Jin L. Y. (2023). Follicle-stimulating hormone orchestrates glucose-stimulated insulin secretion of pancreatic islets. *Nat. Commun.* 14:11. 10.1038/s41467-023-42801-6 37914684 PMC10620214

[B15] Crépieux P. Marion S. Martinat N. Fafeur V. Vern Y. L. Kerboeuf D. (2001). The ERK-dependent signalling is stage-specifically modulated by FSH, during primary Sertoli cell maturation. *Oncogene* 20 4696–4709. 10.1038/sj.onc.1204632 11498792

[B16] Dark H. E. An Y. Duggan M. R. Joynes C. Davatzikos C. Erus G. (2024). Alzheimer’s and neurodegenerative disease biomarkers in blood predict brain atrophy and cognitive decline. *Alzheimers Res. Therapy* 16:11. 10.1186/s13195-024-01459-y 38689358 PMC11059745

[B17] Dratva M. A. Banks S. J. Panizzon M. S. Galasko D. Sundermann E. E. (2024). Low testosterone levels relate to poorer cognitive function in women in an APOE-ε4-dependant manner. *Biol. Sex Dif.* 15:15. 10.1186/s13293-024-00620-4 38835072 PMC11151480

[B18] Du H. Mizokami A. Ni J. Zhang S. Yamawaki Y. Sano T. (2025). Role of testosterone signaling in microglia: A potential role for sex-related differences in Alzheimer’s disease. *Adv. Sci.* 12:e2413375. 10.1002/advs.202413375 40125707 PMC12097063

[B19] Epperson C. N. Sammel M. D. Freeman E. W. (2013). Menopause effects on verbal memory: Findings from a longitudinal community cohort. *J. Clin. Endocrinol. Metab.* 98 3829–3838. 10.1210/jc.2013-1808 23836935 PMC3763981

[B20] Ferretti M. T. Iulita M. F. Cavedo E. Chiesa P. A. Schumacher Dimech A. Santuccione Chadha A. (2018). Sex differences in Alzheimer disease - The gateway to precision medicine. *Nat. Rev. Neurol.* 14 457–469. 10.1038/s41582-018-0032-9 29985474

[B21] Fisher D. W. Bennett D. A. Dong H. (2018). Sexual dimorphism in predisposition to Alzheimer’s disease. *Neurobiol. Aging* 70 308–324. 10.1016/j.neurobiolaging.2018.04.004 29754747 PMC6368179

[B22] Ford A. H. Yeap B. B. Flicker L. Hankey G. J. Chubb S. A. P. Golledge J. (2018). Sex hormones and incident dementia in older men: The health in men study. *Psychoneuroendocrinology* 98 139–147. 10.1016/j.psyneuen.2018.08.013 30144781

[B23] Geerlings M. I. Strozyk D. Masaki K. Remaley A. T. Petrovitch H. Ross G. W. (2006). Endogenous sex hormones, cognitive decline, and future dementia in old men. *Ann. Neurol.* 60 346–355. 10.1002/ana.20918 16865684

[B24] Gillies G. E. McArthur S. (2010). Estrogen actions in the brain and the basis for differential action in men and women: A case for sex-specific medicines. *Pharmacol. Rev.* 62 155–198. 10.1124/pr.109.002071 20392807 PMC2879914

[B25] Goodenough S. Engert S. Behl C. (2000). Testosterone stimulates rapid secretory amyloid precursor protein release from rat hypothalamic cells via the activation of the mitogen-activated protein kinase pathway. *Neurosci. Lett.* 296 49–52. 10.1016/s0304-3940(00)01622-0 11099831

[B26] Gouras G. K. Xu H. Gross R. S. Greenfield J. P. Hai B. Wang R. (2000). Testosterone reduces neuronal secretion of Alzheimer’s beta-amyloid peptides. *Proc. Natl. Acad. Sci. U S A.* 97 1202–1205. 10.1073/pnas.97.3.1202 10655508 PMC15568

[B27] Greendale G. A. Huang M. H. Wight R. G. Seeman T. Luetters C. Avis N. E. (2009). Effects of the menopause transition and hormone use on cognitive performance in midlife women. *Neurology* 72 1850–1857. 10.1212/WNL.0b013e3181a71193 19470968 PMC2690984

[B28] Grimm A. Schmitt K. Lang U. E. Mensah-Nyagan A. G. Eckert A. (2014). Improvement of neuronal bioenergetics by neurosteroids: Implications for age-related neurodegenerative disorders. *Biochim. Biophys. Acta* 1842 2427–2438. 10.1016/j.bbadis.2014.09.013 25281013

[B29] Guo Y. J. Zhao M. Bo T. Ma S. Z. Yuan Z. S. Chen W. B. (2019). Blocking FSH inhibits hepatic cholesterol biosynthesis and reduces serum cholesterol. *Cell Res.* 29 151–166. 10.1038/s41422-018-0123-6 30559440 PMC6355920

[B30] Hammond G. L. Hirvonen J. Vihko R. (1983). Progesterone, androstenedione, testosterone, 5 alpha-dihydrotestosterone and androsterone concentrations in specific regions of the human brain. *J. Steroid Biochem.* 18 185–189. 10.1016/0022-4731(83)90086-9 6843122

[B31] Hansson O. (2021). Biomarkers for neurodegenerative diseases. *Nat. Med.* 27 954–963. 10.1038/s41591-021-01382-x 34083813

[B32] Heijboer A. C. Hannema S. E. (2023). Androgen excess and deficiency: Analytical and diagnostic approaches. *Clin. Chem.* 69 1361–1373. 10.1093/clinchem/hvad146 37794651

[B33] Hua X. Hibar D. P. Ching C. R. Boyle C. P. Rajagopalan P. Gutman B. A. (2013). Unbiased tensor-based morphometry: Improved robustness and sample size estimates for Alzheimer’s disease clinical trials. *Neuroimage* 66 648–661. 10.1016/j.neuroimage.2012.10.086 23153970 PMC3785376

[B34] Huang G. Wharton W. Bhasin S. Harman S. M. Pencina K. M. Tsitouras P. (2016). Effects of long-term testosterone administration on cognition in older men with low or low-to-normal testosterone concentrations: A prespecified secondary analysis of data from the randomised, double-blind, placebo-controlled TEAAM trial. *Lancet Diabetes Endocrinol.* 4 657–665. 10.1016/s2213-8587(16)30102-4 27377542

[B35] Huque H. Eramudugolla R. Chidiac B. Ee N. Ehrenfeld L. Matthews F. E. (2023). Could country-level factors explain sex differences in dementia incidence and prevalence? A systematic review and meta-analysis. *J. Alzheimers Dis.* 91 1231–1241. 10.3233/jad-220724 36565114 PMC9986694

[B36] Jayaraman A. Lent-Schochet D. Pike C. J. (2014). Diet-induced obesity and low testosterone increase neuroinflammation and impair neural function. *J. Neuroinflammation* 11:162. 10.1186/s12974-014-0162-y 25224590 PMC4190446

[B37] Jia J. X. Cui C. L. Yan X. S. Zhang B. F. Song W. Huo D. S. (2016). Effects of testosterone on synaptic plasticity mediated by androgen receptors in male SAMP8 mice. *J. Toxicol. Environ. Health A* 79 849–855. 10.1080/15287394.2016.1193113 27599230

[B38] Jia Y. M. Du X. Z. Wang Y. N. Song Q. H. He L. (2024). Sex differences in luteinizing hormone aggravates Aβ deposition in APP/ PS1 and Aβ1-42-induced mouse models of Alzheimer’s disease. *Eur. J. Pharmacol.* 970:14. 10.1016/j.ejphar.2024.176485 38492878

[B39] Jiang J. W. Jiang T. L. Wang X. H. Zhao M. Shi H. P. Zhang H. Y. (2025). Malnutrition exacerbating neuropsychiatric symptoms on the Alzheimer’s continuum is relevant to the cAMP signaling pathway: Human and mouse studies. *Alzheimers Dement.* 21:19. 10.1002/alz.14506 39868480 PMC11848410

[B40] Judd H. L. Judd G. E. Lucas W. E. Yen S. S. (1974). Endocrine function of the postmenopausal ovary: Concentration of androgens and estrogens in ovarian and peripheral vein blood. *J. Clin. Endocrinol. Metab.* 39 1020–1024. 10.1210/jcem-39-6-1020 4430702

[B41] Koran M. E. I. Wagener M. Hohman T. J. (2017). Sex differences in the association between AD biomarkers and cognitive decline. *Brain Imaging Behav.* 11 205–213. 10.1007/s11682-016-9523-8 26843008 PMC4972701

[B42] Lemprière S. (2022). FSH provides link between menopause and AD. *Nat. Rev. Neurol.* 18 251–251. 10.1038/s41582-022-00647-4 35292744

[B43] Leranth C. Petnehazy O. MacLusky N. J. (2003). Gonadal hormones affect spine synaptic density in the CA1 hippocampal subfield of male rats. *J. Neurosci.* 23 1588–1592. 10.1523/JNEUROSCI.23-05-01588.2003 12629162 PMC6741990

[B44] Liu P. Ji Y. T. Yuen T. Rendina-Ruedy E. DeMambro V. E. Dhawan S. (2017). Blocking FSH induces thermogenic adipose tissue and reduces body fat. *Nature* 546:107. 10.1038/nature22342 28538730 PMC5651981

[B45] Liu X. M. Chan H. C. Ding G. L. Cai J. Song Y. Wang T. T. (2015). FSH regulates fat accumulation and redistribution in aging through the Gai/Ca2+/CREB pathway. *Aging Cell.* 14 409–420. 10.1111/acel.12331 25754247 PMC4406670

[B46] Lopez-Lee C. Torres E. R. S. Carling G. Gan L. (2024). Mechanisms of sex differences in Alzheimer’s disease. *Neuron* 112 1208–1221. 10.1016/j.neuron.2024.01.024 38402606 PMC11076015

[B47] Marriott R. J. Murray K. Flicker L. Hankey G. J. Matsumoto A. M. Dwivedi G. (2022a). Lower serum testosterone concentrations are associated with a higher incidence of dementia in men: The UK Biobank prospective cohort study. *Alzheimers Dement.* 18 1907–1918. 10.1002/alz.12529 34978125

[B48] Marriott R. J. Murray K. Hankey G. J. Manning L. Dwivedi G. Wu F. C. W. (2022b). Longitudinal changes in serum testosterone and sex hormone-binding globulin in men aged 40-69 years from the UK Biobank. *Clin. Endocrinol.* 96 589–598. 10.1111/cen.14648 34873743

[B49] Matyi J. M. Rattinger G. B. Schwartz S. Buhusi M. Tschanz J. T. (2019). Lifetime estrogen exposure and cognition in late life: The Cache county study. *Menopause* 26 1366–1374. 10.1097/gme.0000000000001405 31613825 PMC7448538

[B50] Meethal S. V. Smith M. A. Bowen R. L. Atwood C. S. (2005). The gonadotropin connection in Alzheimer’s disease. *Endocrine* 26 317–326. 26,317 10.1385/endo 16034187

[B51] Meyer P. M. Powell L. H. Wilson R. S. Everson-Rose S. A. Kravitz H. M. Luborsky J. L. (2003). A population-based longitudinal study of cognitive functioning in the menopausal transition. *Neurology* 61 801–806. 10.1212/01.Wnl.0000079051.91602.E2 14504324

[B52] Moffat S. D. Zonderman A. B. Metter E. J. Kawas C. Blackman M. R. Harman S. M. (2004). Free testosterone and risk for Alzheimer disease in older men. *Neurology* 62 188–193. 10.1212/wnl.62.2.188 14745052

[B53] Nebel R. A. Aggarwal N. T. Barnes L. L. Gallagher A. Goldstein J. M. Kantarci K. (2018). Understanding the impact of sex and gender in Alzheimer’s disease: A call to action. *Alzheimers Dement.* 14 1171–1183. 10.1016/j.jalz.2018.04.008 29907423 PMC6400070

[B54] Nelson H. D. Humphrey L. L. Nygren P. Teutsch S. M. Allan J. D. (2002). Postmenopausal hormone replacement therapy: scientific review. *JAMA* 288 872–881. 10.1001/jama.288.7.872 12186605

[B55] O’Brien J. Jackson J. W. Grodstein F. Blacker D. Weuve J. (2014). Postmenopausal hormone therapy is not associated with risk of all-cause dementia and Alzheimer’s disease. *Epidemiol. Rev.* 36 83–103. 10.1093/epirev/mxt008 24042430 PMC3873843

[B56] Oh D. J. Baek K. H. Kang D. W. Hong Y. J. Jeong C. (2025). Association between serum follicle-stimulating hormone levels and cognitive function in middle-aged and older women. *J. Korean Med. Sci.* 40:e15. 10.3346/jkms.2025.40.e15 40098489 PMC11913625

[B57] Palm R. Chang J. Blair J. Garcia-Mesa Y. Lee H. G. Castellani R. J. (2014). Down-regulation of serum gonadotropins but not estrogen replacement improves cognition in aged-ovariectomized 3xTg AD female mice. *J. Neurochem.* 130 115–125. 10.1111/jnc.12706 24601954 PMC4672731

[B58] Park S. Y. Tournell C. Sinjoanu R. C. Ferreira A. (2007). Caspase-3- and calpain-mediated tau cleavage are differentially prevented by estrogen and testosterone in beta-amyloid-treated hippocampal neurons. *Neuroscience* 144 119–127. 10.1016/j.neuroscience.2006.09.012 17055174 PMC1955430

[B59] Pike C. J. (2001). Testosterone attenuates β-amyloid toxicity in cultured hippocampal neurons. *Brain Res.* 919 160–165. 10.1016/s0006-8993(01)03024-4 11689174

[B60] Qi X. Y. Guo Y. J. Song Y. F. Yu C. X. Zhao L. F. Fang L. (2018). Follicle-stimulating hormone enhances hepatic gluconeogenesis by GRK2-mediated AMPK hyperphosphorylation at Ser485 in mice. *Diabetologia* 61 1180–1192. 10.1007/s00125-018-4562-x 29442133

[B61] Quesnel M. J. Labonté A. Picard C. Zetterberg H. Blennow K. Brinkmalm A. (2024). Insulin-like growth factor binding protein-2 in at-risk adults and autopsy-confirmed Alzheimer brains. *Brain* 147 1680–1695. 10.1093/brain/awad398 37992295 PMC11068109

[B62] Randolph J. F. Zheng H. Y. Sowers M. R. Crandall C. Crawford S. Gold E. B. (2011). Change in follicle-stimulating hormone and estradiol across the menopausal transition: Effect of age at the final menstrual period. *J. Clin. Endocrinol. Metab.* 96 746–754. 10.1210/jc.2010-1746 21159842 PMC3047231

[B63] Rosario E. R. Chang L. Head E. H. Stanczyk F. Z. Pike C. J. (2011). Brain levels of sex steroid hormones in men and women during normal aging and in Alzheimer’s disease. *Neurobiol. Aging* 32 604–613. 10.1016/j.neurobiolaging.2009.04.008 19428144 PMC2930132

[B64] Satizabal C. Beiser A. S. Seshadri S. (2016). Incidence of dementia over three decades in the framingham heart study. *N. Engl. J. Med.* 375 93–94. 10.1056/NEJMc1604823 PMC6374770 27406362

[B65] Sauvé F. Kacimi L. Prévot V. (2024). The hypothalamic-pituitary-gonadal axis and the enigma of Alzheimer disease sex differences. *Nat. Rev. Endocrinol.* 20 317–318. 10.1038/s41574-024-00981-1 38514817 PMC7616426

[B66] Schulz K. Korz V. (2010). Hippocampal testosterone relates to reference memory performance and synaptic plasticity in male rats. *Front. Behav. Neurosci.* 4:187. 10.3389/fnbeh.2010.00187 21188275 PMC3006668

[B67] Seidl J. N. T. Massman P. J. (2015). Relationships between testosterone levels and cognition in patients with Alzheimer disease and nondemented elderly men. *J. Geriatr. Psychiatry Neurol.* 28 27–39. 10.1177/0891988714541872 25009157

[B68] Shattuck D. W. Leahy R. M. (2002). BrainSuite: An automated cortical surface identification tool. *Med. Image Anal.* 6 129–142. 10.1016/s1361-8415(02)00054-3 12045000

[B69] Shen Y. Tian M. Zheng Y. Q. Gong F. Fu A. K. Y. Ip N. Y. (2016). Stimulation of the hippocampal POMC/MC4R circuit alleviates synaptic plasticity impairment in an Alzheimer’s disease model. *Cell Rep.* 17 1819–1831. 10.1016/j.celrep.2016.10.043 27829153

[B70] Shi Z. M. Araujo A. B. Martin S. O’Loughlin P. Wittert G. A. (2013). Longitudinal changes in testosterone over five years in community-dwelling men. *J. Clin. Endocrinol. Metab.* 98 3289–3297. 10.1210/jc.2012-3842 23775354

[B71] Short R. A. Bowen R. L. O’Brien P. C. Graff-Radford N. R. (2001). Elevated gonadotropin levels in patients with Alzheimer disease. *Mayo Clin. Proc.* 76 906–909. 10.4065/76.9.906 11560301

[B72] Simoni M. Gromoll J. Nieschlag E. (1997). The follicle-stimulating hormone receptor: Biochemistry, molecular biology, physiology, and pathophysiology. *Endocr. Rev.* 18 739–773. 10.1210/edrv.18.6.0320 9408742

[B73] Song Y. Wang E. S. Xing L. L. Shi S. Qu F. Zhang D. (2016). Follicle-stimulating hormone induces postmenopausal dyslipidemia through inhibiting hepatic cholesterol metabolism. *J. Clin. Endocrinol. Metab.* 101 253–262. 10.1210/jc.2015-2724 26583582

[B74] Tang S. Xiao Z. Lin F. Liang X. Ma X. Wu J. (2024). Joint effect of testosterone and neurofilament light chain on cognitive decline in men: The Shanghai aging study. *Alzheimers Dement.* 20 5290–5298. 10.1002/alz.13889 38837321 PMC11350006

[B75] Verdile G. Laws S. M. Henley D. Ames D. Bush A. I. Ellis K. A. (2014). Associations between gonadotropins, testosterone and β amyloid in men at risk of Alzheimer’s disease. *Mol. Psychiatry* 19 69–75. 10.1038/mp.2012.147 23089633

[B76] Verdile G. Yeap B. B. Clarnette R. M. Dhaliwal S. Burkhardt M. S. Chubb S. A. P. (2008). Luteinizing hormone levels are positively correlated with plasma amyloid-β protein levels in elderly men. *J. Alzheimers Dis.* 14 201–208. 10.3233/jad-2008-14208 18560131

[B77] Viña J. Lloret A. (2010). Why women have more Alzheimer’s disease than men: Gender and mitochondrial toxicity of amyloid-beta peptide. *J. Alzheimers Dis.* 20 S527–S533. 10.3233/jad-2010-100501 20442496

[B78] Xiong J. Kang S. S. Wang M. Wang Z. Xia Y. Liao J. (2023). FSH and ApoE4 contribute to Alzheimer’s disease-like pathogenesis via C/EBPβ/δ-secretase in female mice. *Nat. Commun.* 14:6577. 10.1038/s41467-023-42282-7 37852961 PMC10584868

[B79] Xiong J. Kang S. S. Wang Z. H. Liu X. Kuo T. C. Korkmaz F. (2022). FSH blockade improves cognition in mice with Alzheimer’s disease. *Nature* 603:470. 10.1038/s41586-022-04463-0 35236988 PMC9940301

[B80] Yaffe K. (2004). Testosterone and the brain: Uncharted territory. *Lancet Neurol.* 3 270–270. 10.1016/s1474-4422(04)00732-x 15099540

[B81] Yan X. S. Yang Z. J. Jia J. X. Song W. Fang X. Cai Z. P. (2019). Protective mechanism of testosterone on cognitive impairment in a rat model of Alzheimer’s disease. *Neural Regen. Res.* 14 649–657. 10.4103/1673-5374.245477 30632505 PMC6352583

[B82] Zandi P. P. Carlson M. C. Plassman B. L. Welsh-Bohmer K. A. Mayer L. S. Steffens D. C. (2002). Hormone replacement therapy and incidence of Alzheimer disease in older women: The Cache County Study. *JAMA* 288 2123–2129. 10.1001/jama.288.17.2123 12413371

[B83] Zhu Y. Tu Y. Ren C. Ke Y. Guo Q. (2025). Elevated gonadotropins and risk of dementia in Chinese adults aged over 80: A cross-sectional study. *Front. Aging Neurosci.* 17:1651723. 10.3389/fnagi.2025.1651723 41180813 PMC12575386

